# (1*E*,2*E*)-1,2-Bis[1-(2-methoxy­phen­yl)ethyl­idene]hydrazine^1^

**DOI:** 10.1107/S1600536810043266

**Published:** 2010-10-31

**Authors:** Suchada Chantrapromma, Patcharaporn Jansrisewangwong, Hoong-Kun Fun

**Affiliations:** a Crystal Materials Research Unit, Department of Chemistry, Faculty of Science, Prince of Songkla University, Hat-Yai, Songkhla 90112, Thailand; b X-ray Crystallography Unit, School of Physics, Universiti Sains Malaysia, 11800 USM, Penang, Malaysia

## Abstract

There are two crystallographically independent mol­ecules in the asymmetric unit of the title compound, C_18_H_20_N_2_O_2_. The two mol­ecules exist in an *E*,*E* configuration with respect to the two C=N double bonds. The dihedral angles between the two benzene rings in each mol­ecule are 16.89 (6) and 18.84 (6)°. In each mol­ecule, the two meth­oxy groups are coplanar with their attached benzene rings, with r.m.s. deviations of 0.0078 and 0.0336 Å in one mol­ecule, and 0.0163 and 0.0207 Å in the other. An intra­molecular C—H⋯O hydrogen bond is present in one mol­ecule. In the crystal structure, mol­ecules are arranged into ribbons along the *c* axis. These ribbons are further stacked along the *a* axis. The mol­ecules are consolidated by C⋯N [3.306 (2)–3.427 (2) Å] and C⋯O [3.3284 (16)–3.3863 (15) Å] short contacts. C—H⋯π inter­actions are also observed.

## Related literature

For related structures, see: Jansrisewangwong *et al.* (2010 ▶); Zhao *et al.* (2006 ▶). For background to and biological activities of hydra­zones, see: El-Sherif (2009 ▶); Melnyk *et al.* (2006 ▶); Papakonstanti­nou-Garoufalias *et al.* (2002 ▶); Patole *et al.* (2003 ▶); Sridhar *et al.* (2002 ▶). For bond-length data, see: Allen *et al.* (1987 ▶). For hydrogen-bond motifs, see: Bernstein *et al.* (1995 ▶). For the stability of the temperature controller used in the data collection, see: Cosier & Glazer (1986 ▶).
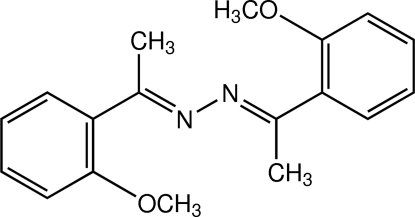


## Experimental

### 

#### Crystal data

C_18_H_20_N_2_O_2_
*M* *_r_* = 296.36
Triclinic, 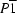

*a* = 7.9695 (2) Å
*b* = 14.8028 (4) Å
*c* = 15.4704 (4) Å
α = 117.909 (1)°
β = 90.151 (1)°
γ = 91.979 (1)°
*V* = 1611.46 (7) Å^3^
*Z* = 4
Mo *K*α radiation
μ = 0.08 mm^−1^
*T* = 100 K
0.55 × 0.37 × 0.20 mm

#### Data collection

Bruker APEXII CCD area-detector diffractometer
Absorption correction: multi-scan (*SADABS*; Bruker, 2005 ▶) *T*_min_ = 0.957, *T*_max_ = 0.984
39016 measured reflections
9367 independent reflections
7773 reflections with *I* > 2σ(*I*)
*R*_int_ = 0.028

#### Refinement

*R*[*F* ^2^ > 2σ(*F* ^2^)] = 0.046
*wR*(*F* ^2^) = 0.123
*S* = 1.02
9367 reflections
405 parameters
H-atom parameters constrained
Δρ_max_ = 0.37 e Å^−3^
Δρ_min_ = −0.24 e Å^−3^

### 

Data collection: *APEX2* (Bruker, 2005 ▶); cell refinement: *SAINT* (Bruker, 2005 ▶); data reduction: *SAINT*; program(s) used to solve structure: *SHELXTL* (Sheldrick, 2008 ▶); program(s) used to refine structure: *SHELXTL*; molecular graphics: *SHELXTL*; software used to prepare material for publication: *SHELXTL* and *PLATON* (Spek, 2009 ▶).

## Supplementary Material

Crystal structure: contains datablocks global, I. DOI: 10.1107/S1600536810043266/rz2497sup1.cif

Structure factors: contains datablocks I. DOI: 10.1107/S1600536810043266/rz2497Isup2.hkl

Additional supplementary materials:  crystallographic information; 3D view; checkCIF report

## Figures and Tables

**Table 1:** Hydrogen-bond geometry (Å, °)

*D*—H⋯*A*	*D*—H	H⋯*A*	*D*⋯*A*	*D*—H⋯*A*
C17*B*—H17*F*⋯O2*B*	0.96	2.36	2.9918 (17)	123
C15*B*—H15*E*⋯*Cg*1^i^	0.96	2.83	3.5974 (17)	138
C18*A*—H18*C*⋯*Cg*2^ii^	0.96	2.90	3.6976 (17)	141

Symmetry codes: (i) 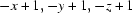; (ii) 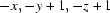. *Cg*1 and *Cg*2 are the centroids of the C9*A*–C14*A* and C1*B*–C6*B* rings, respectively.

**Fig. 1.**
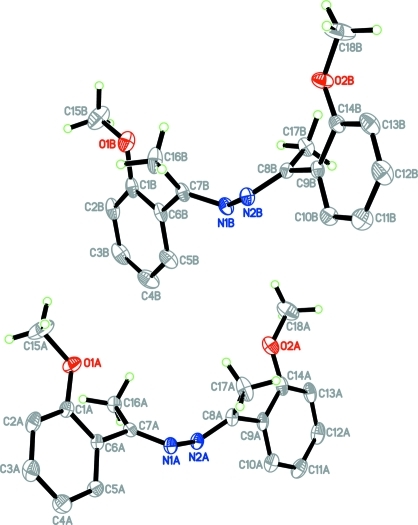
The molecular structure of the title compound, showing 50% probability displacement ellipsoids and the atom-numbering scheme. Aromatic H atoms were omitted for clarify.

**Fig. 2.**
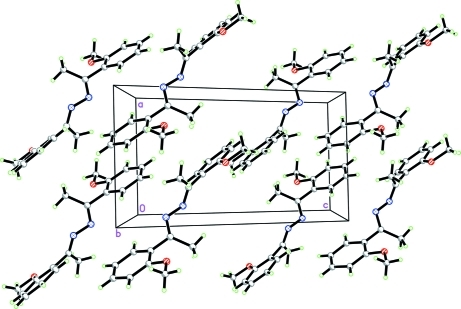
The crystal packing of the title compound viewed along the b axis.

## supplementary crystallographic information

### Comment

Hydrazone derivatives play an important role in antimicrobial activity. Furthermore, a number of hydrazide and hydrazone derivatives have demonstrated to possess antibacterial, antifungal (Papakonstantinou-Garoufalias *et al.*, 2002), anticonvulsant (Sridhar *et al.*, 2002), anti-inflammatory (El-Sherif, 2009), antimalarial (Melnyk *et al.*, 2006) and antituberculosis activities (Patole *et al.*, 2003). These interesting features of hydrazones have led us to synthesize several hydrazone derivatives to study their antibacterial activities. Herein we report the synthesis and crystal structure of the title compound, (I).

There are two molecules (*A* and *B*) in an asymmetric unit of (I) (Fig. 1). The two molecules have slightly different bond angles but exist in the same configuration which is *E*,*E* configuration with respect to the C7═N1 and C8═N2 double bonds [1.2835 (14) and 1.2821 (14) Å, respectively in molecule *A*, and 1.2877 (14) and 1,2837 (13) Å in molecule *B*] and with torsion angles N2–N1–C7–C6 = 169.23 (9)° and N1–N2–C8–C9 = 167.25 (19)° in molecule *A* [-166.13 (9) and -166.24 (9)° in molecule *B*]. The dihedral angle between the two benzene rings is 16.89 (6)° in molecule *A* [18.84 (6)° in molecule *B*]. The two methoxy groups are co-planar with each attached benzene ring with the dihedral angles of C15–O1–C1–C2 = -3.00 (17)° and C18–O2–C14–C13 = 1.85 (15)° in molecule *A* [the corresponding values are 0.99 (16) and -1.54 (18)° in molecule *B*]. The two methyl groups are twisted from the plane of benzene rings and their orientations can be indicated by the torsion angles C1–C6–C7–C16 = -48.69 (14)° and C17–C8–C9–C14 = -49.33 (16)° in molecule *A* [the correspondibng values are 44.04 (16) and 53.67 (15)° in molecule *B*]. In molecule *B*, the intramolecular C17B—H17F···O2B weak interaction (Table 1) generate S(6) ring motif (Bernstein *et al.*, 1995). The bond distances are of normal values (Allen *et al.*, 1987) and are comparable with a related structure (Jansrisewangwong *et al.*, 2010; Zhao *et al.*, 2006).

In the crystal structure (Fig. 2), the molecules are arranged into ribbons along the *c* axis. These ribbons are further stacked along the *a* axis. The molecules are consolidated by C···N [3.306 (2)–3.427 (2) Å] and C···O [3.3284 (16)–3.3863 (15) Å] short contacts. C—H···π interactions were also observed (Table 1); *Cg*_1_ and *Cg*_2_ are the centroid of C9A–C14A and C1B–C6B rings, respectively.

### Experimental

The title compound was synthesized by mixing a solution (1:2 molar ratio) of hydrazine hydrate (0.10 ml, 2 mmol) and 2-methoxyacetophenone (0.55 ml, 4 mmol) in ethanol (20 ml). The resulting solution was refluxed for 5 h, yielding the yellow crystalline solid. The resultant solid was filtered off and washed with methanol. Yellow block-shaped single crystals of the title compound suitable for *x*-ray structure determination were recrystalized from acetone by slow evaporation of the solvent at room temperature over several days.

### Refinement

H atoms were positioned geometrically and allowed to ride on their parent atoms, with d(C—H) = 0.93 Å for aromatic and 0.96 Å for CH_3_ atoms. The *U*_iso_ values were constrained to be 1.5*U*_eq_ of the carrier atom for methyl H atoms and 1.2*U*_eq_ for the remaining H atoms. A rotating group model was used for the methyl groups. The highest residual electron density peak is located at 0.69 Å from C7A and the deepest hole is located at 0.24 Å from H17D.

### Crystal data

**Table:**

C_18_H_20_N_2_O_2_	*Z* = 4
*M**_r_* = 296.36	*F*(000) = 632
Triclinic, *P*1	*D*_x_ = 1.222 Mg m^−^^3^
Hall symbol: -P 1	Mo *K*α radiation, λ = 0.71073 Å
*a* = 7.9695 (2) Å	Cell parameters from 9367 reflections
*b* = 14.8028 (4) Å	θ = 1.5–30.0°
*c* = 15.4704 (4) Å	µ = 0.08 mm^−^^1^
α = 117.909 (1)°	*T* = 100 K
β = 90.151 (1)°	Block, yellow
γ = 91.979 (1)°	0.55 × 0.37 × 0.20 mm
*V* = 1611.46 (7) Å^3^	

### Data collection

**Table:**

Bruker APEXII CCD area-detector diffractometer	9367 independent reflections
Radiation source: sealed tube	7773 reflections with *I* > 2σ(*I*)
graphite	*R*_int_ = 0.028
φ and ω scans	θ_max_ = 30.0°, θ_min_ = 1.5°
Absorption correction: multi-scan (*SADABS*; Bruker, 2005)	*h* = −11→11
*T*_min_ = 0.957, *T*_max_ = 0.984	*k* = −20→20
39016 measured reflections	*l* = −21→21

### Refinement

**Table:**

Refinement on *F*^2^	Primary atom site location: structure-invariant direct methods
Least-squares matrix: full	Secondary atom site location: difference Fourier map
*R*[*F*^2^ > 2σ(*F*^2^)] = 0.046	Hydrogen site location: inferred from neighbouring sites
*wR*(*F*^2^) = 0.123	H-atom parameters constrained
*S* = 1.02	*w* = 1/[σ^2^(*F*_o_^2^) + (0.0567*P*)^2^ + 0.6198*P*] where *P* = (*F*_o_^2^ + 2*F*_c_^2^)/3
9367 reflections	(Δ/σ)_max_ = 0.001
405 parameters	Δρ_max_ = 0.37 e Å^−^^3^
0 restraints	Δρ_min_ = −0.24 e Å^−^^3^

### Special details

**Table:**

Experimental. The crystal was placed in the cold stream of an Oxford Cryosystems Cobra open-flow nitrogen cryostat (Cosier & Glazer, 1986) operating at 120.0 (1) K.
Geometry. All e.s.d.'s (except the e.s.d. in the dihedral angle between two l.s. planes) are estimated using the full covariance matrix. The cell e.s.d.'s are taken into account individually in the estimation of e.s.d.'s in distances, angles and torsion angles; correlations between e.s.d.'s in cell parameters are only used when they are defined by crystal symmetry. An approximate (isotropic) treatment of cell e.s.d.'s is used for estimating e.s.d.'s involving l.s. planes.
Refinement. Refinement of *F*^2^ against ALL reflections. The weighted *R*-factor *wR* and goodness of fit *S* are based on *F*^2^, conventional *R*-factors *R* are based on *F*, with *F* set to zero for negative *F*^2^. The threshold expression of *F*^2^ > σ(*F*^2^) is used only for calculating *R*-factors(gt) *etc*. and is not relevant to the choice of reflections for refinement. *R*-factors based on *F*^2^ are statistically about twice as large as those based on *F*, and *R*- factors based on ALL data will be even larger.

### Fractional atomic coordinates and isotropic or equivalent isotropic displacement parameters (Å^2^)

**Table:**

	*x*	*y*	*z*	*U*_iso_*/*U*_eq_	
O1A	0.75936 (11)	0.36403 (6)	0.20160 (6)	0.02473 (18)	
O2A	0.05444 (11)	0.30674 (7)	0.43253 (6)	0.02626 (18)	
N1A	0.46008 (11)	0.14041 (7)	0.17196 (6)	0.01854 (18)	
N2A	0.37331 (11)	0.12913 (7)	0.24477 (7)	0.01884 (18)	
C1A	0.78882 (13)	0.27395 (9)	0.12023 (8)	0.0191 (2)	
C2A	0.88899 (15)	0.26604 (10)	0.04349 (9)	0.0252 (2)	
H2AA	0.9423	0.3241	0.0460	0.030*	
C3A	0.90873 (16)	0.17096 (11)	−0.03661 (9)	0.0293 (3)	
H3AA	0.9765	0.1655	−0.0875	0.035*	
C4A	0.82876 (17)	0.08417 (10)	−0.04166 (9)	0.0288 (3)	
H4AA	0.8434	0.0206	−0.0953	0.035*	
C5A	0.72626 (14)	0.09266 (9)	0.03416 (8)	0.0217 (2)	
H5AA	0.6701	0.0346	0.0299	0.026*	
C6A	0.70634 (12)	0.18658 (8)	0.11623 (7)	0.01635 (19)	
C7A	0.60121 (13)	0.19120 (8)	0.19741 (7)	0.01595 (19)	
C8A	0.22915 (13)	0.16892 (9)	0.26668 (8)	0.0194 (2)	
C9A	0.12412 (13)	0.13672 (9)	0.32803 (8)	0.0201 (2)	
C10A	0.11483 (15)	0.03358 (10)	0.30463 (8)	0.0251 (2)	
H10A	0.1765	−0.0125	0.2527	0.030*	
C11A	0.01548 (16)	−0.00223 (11)	0.35703 (9)	0.0297 (3)	
H11A	0.0111	−0.0713	0.3404	0.036*	
C12A	−0.07677 (15)	0.06652 (11)	0.43420 (9)	0.0295 (3)	
H12A	−0.1452	0.0431	0.4687	0.035*	
C13A	−0.06803 (14)	0.16999 (11)	0.46046 (9)	0.0261 (2)	
H13A	−0.1295	0.2156	0.5128	0.031*	
C14A	0.03324 (13)	0.20564 (10)	0.40820 (8)	0.0217 (2)	
C15A	0.8430 (2)	0.45433 (11)	0.20980 (12)	0.0457 (4)	
H15A	0.8164	0.5115	0.2710	0.069*	
H15B	0.8070	0.4664	0.1570	0.069*	
H15C	0.9621	0.4460	0.2069	0.069*	
C16A	0.66645 (14)	0.24582 (9)	0.30146 (8)	0.0200 (2)	
H16A	0.6653	0.1990	0.3283	0.030*	
H16B	0.5964	0.3018	0.3395	0.030*	
H16C	0.7793	0.2715	0.3030	0.030*	
C17A	0.15991 (16)	0.23508 (12)	0.22654 (10)	0.0323 (3)	
H17A	0.1805	0.2051	0.1577	0.048*	
H17B	0.2138	0.3020	0.2592	0.048*	
H17C	0.0412	0.2404	0.2370	0.048*	
C18A	−0.0294 (2)	0.37775 (12)	0.51799 (10)	0.0364 (3)	
H18A	0.0057	0.4463	0.5331	0.055*	
H18B	−0.0015	0.3658	0.5721	0.055*	
H18C	−0.1486	0.3687	0.5061	0.055*	
O1B	0.45456 (11)	0.82808 (7)	0.44885 (6)	0.02682 (19)	
O2B	−0.27309 (11)	0.85346 (6)	0.18823 (6)	0.02520 (18)	
N1B	0.11694 (11)	0.64386 (7)	0.25137 (7)	0.01901 (18)	
N2B	0.03346 (11)	0.65363 (7)	0.17709 (7)	0.01845 (18)	
C1B	0.46224 (13)	0.72673 (10)	0.42254 (8)	0.0221 (2)	
C2B	0.55967 (14)	0.68929 (11)	0.47331 (9)	0.0259 (2)	
H2BA	0.6262	0.7344	0.5266	0.031*	
C3B	0.55744 (16)	0.58569 (11)	0.44459 (9)	0.0311 (3)	
H3BA	0.6235	0.5615	0.4784	0.037*	
C4B	0.45791 (18)	0.51747 (11)	0.36600 (10)	0.0323 (3)	
H4BA	0.4555	0.4479	0.3474	0.039*	
C5B	0.36140 (15)	0.55442 (10)	0.31511 (9)	0.0262 (2)	
H5BA	0.2944	0.5087	0.2624	0.031*	
C6B	0.36273 (13)	0.65829 (9)	0.34121 (8)	0.0204 (2)	
C7B	0.26160 (13)	0.69058 (9)	0.27974 (8)	0.0192 (2)	
C8B	−0.11241 (12)	0.69174 (8)	0.19615 (7)	0.01565 (19)	
C9B	−0.21463 (12)	0.67896 (8)	0.10995 (7)	0.01684 (19)	
C10B	−0.23044 (14)	0.58243 (9)	0.02918 (8)	0.0219 (2)	
H10B	−0.1732	0.5287	0.0291	0.026*	
C11B	−0.33037 (16)	0.56475 (10)	−0.05160 (9)	0.0290 (3)	
H11B	−0.3420	0.4995	−0.1045	0.035*	
C12B	−0.41238 (16)	0.64566 (11)	−0.05217 (9)	0.0313 (3)	
H12B	−0.4786	0.6344	−0.1061	0.038*	
C13B	−0.39686 (15)	0.74310 (10)	0.02671 (9)	0.0274 (3)	
H13B	−0.4515	0.7969	0.0253	0.033*	
C14B	−0.29881 (13)	0.75991 (9)	0.10809 (8)	0.0196 (2)	
C15B	0.5555 (2)	0.89710 (12)	0.53241 (11)	0.0390 (3)	
H15D	0.5325	0.9664	0.5484	0.058*	
H15E	0.6722	0.8856	0.5176	0.058*	
H15F	0.5293	0.8855	0.5870	0.058*	
C16B	0.33209 (15)	0.76471 (12)	0.24739 (10)	0.0311 (3)	
H16D	0.3033	0.7408	0.1797	0.047*	
H16E	0.4521	0.7703	0.2558	0.047*	
H16F	0.2861	0.8305	0.2860	0.047*	
C17B	−0.18961 (14)	0.73647 (9)	0.29538 (8)	0.0216 (2)	
H17D	−0.1033	0.7526	0.3444	0.032*	
H17E	−0.2696	0.6877	0.2978	0.032*	
H17F	−0.2453	0.7976	0.3074	0.032*	
C18B	−0.3482 (2)	0.93885 (11)	0.18693 (12)	0.0457 (4)	
H18D	−0.3197	0.9997	0.2466	0.069*	
H18E	−0.4680	0.9277	0.1809	0.069*	
H18F	−0.3074	0.9467	0.1324	0.069*	

### Atomic displacement parameters (Å^2^)

**Table:**

	*U*^11^	*U*^22^	*U*^33^	*U*^12^	*U*^13^	*U*^23^
O1A	0.0321 (4)	0.0190 (4)	0.0229 (4)	−0.0043 (3)	0.0011 (3)	0.0100 (3)
O2A	0.0248 (4)	0.0347 (5)	0.0257 (4)	0.0080 (3)	0.0060 (3)	0.0190 (4)
N1A	0.0171 (4)	0.0241 (5)	0.0169 (4)	0.0001 (3)	0.0025 (3)	0.0117 (4)
N2A	0.0171 (4)	0.0243 (5)	0.0175 (4)	−0.0023 (3)	0.0010 (3)	0.0120 (4)
C1A	0.0180 (5)	0.0228 (5)	0.0178 (5)	−0.0019 (4)	−0.0019 (4)	0.0109 (4)
C2A	0.0238 (5)	0.0316 (6)	0.0239 (5)	−0.0085 (5)	−0.0002 (4)	0.0167 (5)
C3A	0.0278 (6)	0.0402 (7)	0.0209 (5)	−0.0039 (5)	0.0068 (4)	0.0155 (5)
C4A	0.0332 (6)	0.0304 (6)	0.0192 (5)	−0.0004 (5)	0.0076 (5)	0.0086 (5)
C5A	0.0242 (5)	0.0225 (5)	0.0186 (5)	−0.0013 (4)	0.0029 (4)	0.0098 (4)
C6A	0.0143 (4)	0.0211 (5)	0.0159 (4)	−0.0002 (4)	−0.0001 (3)	0.0105 (4)
C7A	0.0160 (4)	0.0183 (5)	0.0159 (4)	0.0028 (4)	0.0020 (3)	0.0098 (4)
C8A	0.0160 (4)	0.0285 (6)	0.0170 (5)	−0.0019 (4)	−0.0010 (4)	0.0136 (4)
C9A	0.0138 (4)	0.0330 (6)	0.0178 (5)	−0.0029 (4)	−0.0017 (3)	0.0156 (4)
C10A	0.0235 (5)	0.0328 (6)	0.0191 (5)	−0.0060 (5)	−0.0015 (4)	0.0128 (5)
C11A	0.0303 (6)	0.0353 (7)	0.0271 (6)	−0.0111 (5)	−0.0030 (5)	0.0185 (5)
C12A	0.0226 (5)	0.0480 (8)	0.0264 (6)	−0.0080 (5)	−0.0009 (4)	0.0250 (6)
C13A	0.0166 (5)	0.0458 (7)	0.0221 (5)	0.0020 (5)	0.0026 (4)	0.0209 (5)
C14A	0.0146 (4)	0.0357 (6)	0.0211 (5)	0.0014 (4)	−0.0012 (4)	0.0184 (5)
C15A	0.0716 (11)	0.0246 (7)	0.0364 (8)	−0.0172 (7)	0.0054 (7)	0.0116 (6)
C16A	0.0192 (5)	0.0259 (5)	0.0167 (5)	−0.0003 (4)	−0.0003 (4)	0.0115 (4)
C17A	0.0230 (6)	0.0553 (9)	0.0363 (7)	0.0105 (5)	0.0073 (5)	0.0355 (7)
C18A	0.0446 (8)	0.0412 (8)	0.0298 (6)	0.0173 (6)	0.0119 (6)	0.0207 (6)
O1B	0.0247 (4)	0.0356 (5)	0.0264 (4)	−0.0053 (3)	−0.0075 (3)	0.0201 (4)
O2B	0.0325 (4)	0.0206 (4)	0.0231 (4)	0.0069 (3)	−0.0027 (3)	0.0103 (3)
N1B	0.0166 (4)	0.0256 (5)	0.0178 (4)	0.0042 (3)	−0.0004 (3)	0.0124 (4)
N2B	0.0162 (4)	0.0246 (5)	0.0176 (4)	0.0013 (3)	−0.0015 (3)	0.0124 (4)
C1B	0.0136 (4)	0.0387 (6)	0.0210 (5)	0.0017 (4)	0.0019 (4)	0.0197 (5)
C2B	0.0162 (5)	0.0473 (7)	0.0215 (5)	0.0029 (5)	−0.0004 (4)	0.0220 (5)
C3B	0.0276 (6)	0.0490 (8)	0.0256 (6)	0.0146 (5)	0.0020 (5)	0.0241 (6)
C4B	0.0372 (7)	0.0367 (7)	0.0278 (6)	0.0158 (6)	0.0020 (5)	0.0182 (5)
C5B	0.0261 (6)	0.0343 (6)	0.0202 (5)	0.0103 (5)	0.0016 (4)	0.0138 (5)
C6B	0.0134 (4)	0.0346 (6)	0.0180 (5)	0.0057 (4)	0.0027 (4)	0.0160 (4)
C7B	0.0144 (4)	0.0299 (6)	0.0172 (5)	0.0042 (4)	0.0014 (3)	0.0141 (4)
C8B	0.0150 (4)	0.0179 (5)	0.0161 (4)	−0.0010 (4)	−0.0012 (3)	0.0098 (4)
C9B	0.0134 (4)	0.0228 (5)	0.0168 (4)	0.0009 (4)	−0.0005 (3)	0.0113 (4)
C10B	0.0230 (5)	0.0244 (5)	0.0181 (5)	0.0027 (4)	−0.0012 (4)	0.0097 (4)
C11B	0.0328 (6)	0.0310 (6)	0.0184 (5)	0.0019 (5)	−0.0058 (4)	0.0076 (5)
C12B	0.0299 (6)	0.0427 (7)	0.0203 (5)	0.0069 (5)	−0.0065 (4)	0.0135 (5)
C13B	0.0263 (6)	0.0357 (7)	0.0234 (5)	0.0100 (5)	−0.0021 (4)	0.0160 (5)
C14B	0.0182 (5)	0.0239 (5)	0.0183 (5)	0.0035 (4)	0.0009 (4)	0.0112 (4)
C15B	0.0461 (8)	0.0438 (8)	0.0347 (7)	−0.0164 (6)	−0.0191 (6)	0.0262 (7)
C16B	0.0189 (5)	0.0542 (8)	0.0363 (7)	−0.0069 (5)	−0.0063 (5)	0.0354 (7)
C17B	0.0206 (5)	0.0295 (6)	0.0180 (5)	0.0055 (4)	0.0025 (4)	0.0135 (4)
C18B	0.0700 (11)	0.0272 (7)	0.0393 (8)	0.0196 (7)	−0.0090 (7)	0.0140 (6)

### Geometric parameters (Å, °)

**Table:**

O1A—C1A	1.3673 (14)	O1B—C1B	1.3619 (15)
O1A—C15A	1.4237 (16)	O1B—C15B	1.4366 (16)
O2A—C14A	1.3656 (15)	O2B—C14B	1.3670 (14)
O2A—C18A	1.4325 (15)	O2B—C18B	1.4255 (15)
N1A—C7A	1.2835 (14)	N1B—C7B	1.2877 (14)
N1A—N2A	1.3954 (12)	N1B—N2B	1.3938 (12)
N2A—C8A	1.2821 (14)	N2B—C8B	1.2837 (13)
C1A—C2A	1.3935 (15)	C1B—C2B	1.4004 (15)
C1A—C6A	1.4053 (15)	C1B—C6B	1.4086 (16)
C2A—C3A	1.3875 (18)	C2B—C3B	1.382 (2)
C2A—H2AA	0.9300	C2B—H2BA	0.9300
C3A—C4A	1.3828 (19)	C3B—C4B	1.385 (2)
C3A—H3AA	0.9300	C3B—H3BA	0.9300
C4A—C5A	1.3904 (16)	C4B—C5B	1.3926 (16)
C4A—H4AA	0.9300	C4B—H4BA	0.9300
C5A—C6A	1.3906 (15)	C5B—C6B	1.3946 (18)
C5A—H5AA	0.9300	C5B—H5BA	0.9300
C6A—C7A	1.4876 (14)	C6B—C7B	1.4935 (14)
C7A—C16A	1.5044 (14)	C7B—C16B	1.4977 (17)
C8A—C9A	1.4929 (15)	C8B—C9B	1.4917 (13)
C8A—C17A	1.5013 (16)	C8B—C17B	1.5018 (14)
C9A—C10A	1.3935 (17)	C9B—C10B	1.3907 (15)
C9A—C14A	1.4052 (16)	C9B—C14B	1.4052 (15)
C10A—C11A	1.3936 (17)	C10B—C11B	1.3924 (15)
C10A—H10A	0.9300	C10B—H10B	0.9300
C11A—C12A	1.3858 (19)	C11B—C12B	1.3876 (18)
C11A—H11A	0.9300	C11B—H11B	0.9300
C12A—C13A	1.388 (2)	C12B—C13B	1.3870 (18)
C12A—H12A	0.9300	C12B—H12B	0.9300
C13A—C14A	1.3990 (16)	C13B—C14B	1.3948 (15)
C13A—H13A	0.9300	C13B—H13B	0.9300
C15A—H15A	0.9600	C15B—H15D	0.9600
C15A—H15B	0.9600	C15B—H15E	0.9600
C15A—H15C	0.9600	C15B—H15F	0.9600
C16A—H16A	0.9600	C16B—H16D	0.9600
C16A—H16B	0.9600	C16B—H16E	0.9600
C16A—H16C	0.9600	C16B—H16F	0.9600
C17A—H17A	0.9600	C17B—H17D	0.9600
C17A—H17B	0.9600	C17B—H17E	0.9600
C17A—H17C	0.9600	C17B—H17F	0.9600
C18A—H18A	0.9600	C18B—H18D	0.9600
C18A—H18B	0.9600	C18B—H18E	0.9600
C18A—H18C	0.9600	C18B—H18F	0.9600
			
C1A—O1A—C15A	117.43 (10)	C1B—O1B—C15B	116.31 (10)
C14A—O2A—C18A	116.57 (10)	C14B—O2B—C18B	117.64 (10)
C7A—N1A—N2A	116.67 (9)	C7B—N1B—N2B	116.99 (9)
C8A—N2A—N1A	116.75 (9)	C8B—N2B—N1B	117.11 (9)
O1A—C1A—C2A	124.13 (10)	O1B—C1B—C2B	123.31 (11)
O1A—C1A—C6A	115.45 (9)	O1B—C1B—C6B	116.88 (9)
C2A—C1A—C6A	120.40 (10)	C2B—C1B—C6B	119.79 (12)
C3A—C2A—C1A	119.57 (11)	C3B—C2B—C1B	120.34 (12)
C3A—C2A—H2AA	120.2	C3B—C2B—H2BA	119.8
C1A—C2A—H2AA	120.2	C1B—C2B—H2BA	119.8
C4A—C3A—C2A	120.79 (11)	C2B—C3B—C4B	120.68 (11)
C4A—C3A—H3AA	119.6	C2B—C3B—H3BA	119.7
C2A—C3A—H3AA	119.6	C4B—C3B—H3BA	119.7
C3A—C4A—C5A	119.47 (11)	C3B—C4B—C5B	119.11 (13)
C3A—C4A—H4AA	120.3	C3B—C4B—H4BA	120.4
C5A—C4A—H4AA	120.3	C5B—C4B—H4BA	120.4
C4A—C5A—C6A	121.10 (11)	C4B—C5B—C6B	121.66 (12)
C4A—C5A—H5AA	119.4	C4B—C5B—H5BA	119.2
C6A—C5A—H5AA	119.4	C6B—C5B—H5BA	119.2
C5A—C6A—C1A	118.64 (10)	C5B—C6B—C1B	118.39 (10)
C5A—C6A—C7A	119.18 (10)	C5B—C6B—C7B	118.06 (10)
C1A—C6A—C7A	122.16 (9)	C1B—C6B—C7B	123.53 (11)
N1A—C7A—C6A	115.93 (9)	N1B—C7B—C6B	114.65 (10)
N1A—C7A—C16A	123.32 (9)	N1B—C7B—C16B	123.60 (9)
C6A—C7A—C16A	120.57 (9)	C6B—C7B—C16B	121.53 (9)
N2A—C8A—C9A	115.31 (10)	N2B—C8B—C9B	115.69 (9)
N2A—C8A—C17A	123.59 (10)	N2B—C8B—C17B	124.37 (9)
C9A—C8A—C17A	120.83 (9)	C9B—C8B—C17B	119.59 (9)
C10A—C9A—C14A	118.35 (10)	C10B—C9B—C14B	118.76 (9)
C10A—C9A—C8A	118.65 (10)	C10B—C9B—C8B	118.58 (9)
C14A—C9A—C8A	123.00 (11)	C14B—C9B—C8B	122.64 (10)
C9A—C10A—C11A	121.69 (12)	C9B—C10B—C11B	121.21 (11)
C9A—C10A—H10A	119.2	C9B—C10B—H10B	119.4
C11A—C10A—H10A	119.2	C11B—C10B—H10B	119.4
C12A—C11A—C10A	119.09 (13)	C12B—C11B—C10B	119.19 (11)
C12A—C11A—H11A	120.5	C12B—C11B—H11B	120.4
C10A—C11A—H11A	120.5	C10B—C11B—H11B	120.4
C11A—C12A—C13A	120.65 (11)	C13B—C12B—C11B	120.86 (11)
C11A—C12A—H12A	119.7	C13B—C12B—H12B	119.6
C13A—C12A—H12A	119.7	C11B—C12B—H12B	119.6
C12A—C13A—C14A	120.00 (11)	C12B—C13B—C14B	119.64 (11)
C12A—C13A—H13A	120.0	C12B—C13B—H13B	120.2
C14A—C13A—H13A	120.0	C14B—C13B—H13B	120.2
O2A—C14A—C13A	123.34 (11)	O2B—C14B—C13B	123.88 (10)
O2A—C14A—C9A	116.44 (10)	O2B—C14B—C9B	115.77 (9)
C13A—C14A—C9A	120.19 (12)	C13B—C14B—C9B	120.31 (11)
O1A—C15A—H15A	109.5	O1B—C15B—H15D	109.5
O1A—C15A—H15B	109.5	O1B—C15B—H15E	109.5
H15A—C15A—H15B	109.5	H15D—C15B—H15E	109.5
O1A—C15A—H15C	109.5	O1B—C15B—H15F	109.5
H15A—C15A—H15C	109.5	H15D—C15B—H15F	109.5
H15B—C15A—H15C	109.5	H15E—C15B—H15F	109.5
C7A—C16A—H16A	109.5	C7B—C16B—H16D	109.5
C7A—C16A—H16B	109.5	C7B—C16B—H16E	109.5
H16A—C16A—H16B	109.5	H16D—C16B—H16E	109.5
C7A—C16A—H16C	109.5	C7B—C16B—H16F	109.5
H16A—C16A—H16C	109.5	H16D—C16B—H16F	109.5
H16B—C16A—H16C	109.5	H16E—C16B—H16F	109.5
C8A—C17A—H17A	109.5	C8B—C17B—H17D	109.5
C8A—C17A—H17B	109.5	C8B—C17B—H17E	109.5
H17A—C17A—H17B	109.5	H17D—C17B—H17E	109.5
C8A—C17A—H17C	109.5	C8B—C17B—H17F	109.5
H17A—C17A—H17C	109.5	H17D—C17B—H17F	109.5
H17B—C17A—H17C	109.5	H17E—C17B—H17F	109.5
O2A—C18A—H18A	109.5	O2B—C18B—H18D	109.5
O2A—C18A—H18B	109.5	O2B—C18B—H18E	109.5
H18A—C18A—H18B	109.5	H18D—C18B—H18E	109.5
O2A—C18A—H18C	109.5	O2B—C18B—H18F	109.5
H18A—C18A—H18C	109.5	H18D—C18B—H18F	109.5
H18B—C18A—H18C	109.5	H18E—C18B—H18F	109.5
			
C7A—N1A—N2A—C8A	117.39 (11)	C7B—N1B—N2B—C8B	−121.46 (11)
C15A—O1A—C1A—C2A	−3.00 (17)	C15B—O1B—C1B—C2B	0.99 (16)
C15A—O1A—C1A—C6A	178.73 (12)	C15B—O1B—C1B—C6B	179.48 (11)
O1A—C1A—C2A—C3A	−179.01 (11)	O1B—C1B—C2B—C3B	177.84 (11)
C6A—C1A—C2A—C3A	−0.82 (17)	C6B—C1B—C2B—C3B	−0.61 (16)
C1A—C2A—C3A—C4A	0.69 (19)	C1B—C2B—C3B—C4B	−0.64 (18)
C2A—C3A—C4A—C5A	0.6 (2)	C2B—C3B—C4B—C5B	0.9 (2)
C3A—C4A—C5A—C6A	−1.75 (19)	C3B—C4B—C5B—C6B	0.11 (19)
C4A—C5A—C6A—C1A	1.61 (16)	C4B—C5B—C6B—C1B	−1.33 (17)
C4A—C5A—C6A—C7A	−177.40 (10)	C4B—C5B—C6B—C7B	177.19 (11)
O1A—C1A—C6A—C5A	178.03 (9)	O1B—C1B—C6B—C5B	−176.99 (10)
C2A—C1A—C6A—C5A	−0.31 (15)	C2B—C1B—C6B—C5B	1.56 (16)
O1A—C1A—C6A—C7A	−2.99 (14)	O1B—C1B—C6B—C7B	4.58 (15)
C2A—C1A—C6A—C7A	178.67 (10)	C2B—C1B—C6B—C7B	−176.86 (10)
N2A—N1A—C7A—C6A	169.23 (9)	N2B—N1B—C7B—C6B	−166.13 (9)
N2A—N1A—C7A—C16A	−5.92 (15)	N2B—N1B—C7B—C16B	8.60 (16)
C5A—C6A—C7A—N1A	−45.02 (14)	C5B—C6B—C7B—N1B	40.45 (14)
C1A—C6A—C7A—N1A	136.01 (11)	C1B—C6B—C7B—N1B	−141.12 (11)
C5A—C6A—C7A—C16A	130.28 (11)	C5B—C6B—C7B—C16B	−134.40 (12)
C1A—C6A—C7A—C16A	−48.69 (14)	C1B—C6B—C7B—C16B	44.04 (16)
N1A—N2A—C8A—C9A	167.25 (9)	N1B—N2B—C8B—C9B	−166.24 (9)
N1A—N2A—C8A—C17A	−6.81 (17)	N1B—N2B—C8B—C17B	6.97 (16)
N2A—C8A—C9A—C10A	−43.91 (14)	N2B—C8B—C9B—C10B	48.84 (14)
C17A—C8A—C9A—C10A	130.33 (12)	C17B—C8B—C9B—C10B	−124.72 (11)
N2A—C8A—C9A—C14A	136.44 (11)	N2B—C8B—C9B—C14B	−132.77 (11)
C17A—C8A—C9A—C14A	−49.33 (16)	C17B—C8B—C9B—C14B	53.67 (15)
C14A—C9A—C10A—C11A	1.51 (16)	C14B—C9B—C10B—C11B	−1.41 (17)
C8A—C9A—C10A—C11A	−178.16 (10)	C8B—C9B—C10B—C11B	177.04 (11)
C9A—C10A—C11A—C12A	0.19 (18)	C9B—C10B—C11B—C12B	1.50 (19)
C10A—C11A—C12A—C13A	−1.31 (18)	C10B—C11B—C12B—C13B	−0.5 (2)
C11A—C12A—C13A—C14A	0.68 (18)	C11B—C12B—C13B—C14B	−0.6 (2)
C18A—O2A—C14A—C13A	1.85 (15)	C18B—O2B—C14B—C13B	−1.54 (18)
C18A—O2A—C14A—C9A	−176.08 (10)	C18B—O2B—C14B—C9B	176.41 (12)
C12A—C13A—C14A—O2A	−176.79 (10)	C12B—C13B—C14B—O2B	178.53 (12)
C12A—C13A—C14A—C9A	1.07 (16)	C12B—C13B—C14B—C9B	0.67 (18)
C10A—C9A—C14A—O2A	175.87 (9)	C10B—C9B—C14B—O2B	−177.71 (10)
C8A—C9A—C14A—O2A	−4.47 (15)	C8B—C9B—C14B—O2B	3.90 (15)
C10A—C9A—C14A—C13A	−2.13 (15)	C10B—C9B—C14B—C13B	0.32 (16)
C8A—C9A—C14A—C13A	177.52 (10)	C8B—C9B—C14B—C13B	−178.07 (10)

### Hydrogen-bond geometry (Å, °)

**Table:**

*D*—H···*A*	*D*—H	H···*A*	*D*···*A*	*D*—H···*A*
C17B—H17F···O2B	0.96	2.36	2.9918 (17)	123
C15B—H15E···Cg1^i^	0.96	2.83	3.5974 (17)	138
C18A—H18C···Cg2^ii^	0.96	2.90	3.6976 (17)	141

Symmetry codes:  (i) −*x*+1, −*y*+1, −*z*+1; (ii) −*x*, −*y*+1, −*z*+1.
